# Current Applications of Dynamic Navigation System in Endodontics: A Scoping Review

**DOI:** 10.1055/s-0042-1749361

**Published:** 2022-08-31

**Authors:** Frederico Canato Martinho, Ina Laurie Griffin, Bruna Jordão Motta Corazza

**Affiliations:** 1 Division of Endodontics, Department of Advanced Oral Sciences and Therapeutics, University of Maryland, School of Dentistry, Baltimore, Maryland; 2 Division of Endodontics, Department of Restorative Dentistry, São Paulo State University, Institute of Science and Technology, São José dos Campos, São Paulo, Brazil

**Keywords:** DNS, endodontics, guided, root canal

## Abstract

This scoping review (SCR) was conducted to map the existing literature on dynamic navigation system (DNS), to examine the extent, range, and nature of research activity. Additionally, this SCR disseminates research findings, determines the value of conducting a full systematic review with meta-analysis, and identifies gaps in the existing literature and future directions. This SCR followed Arksey and O'Malley's five stages framework. The electronic search was performed in PubMed (Medline), Scopus (Elsevier), and Web of Science (Clarivate Analytics) databases using a search strategy. Five themes emerged during the descriptive analysis that captured the DNS application in endodontics. The DNS has been explored for creating access cavities (8/18, 44.44%), locating calcified canals (4/18, 22.2%), microsurgery (3/18, 16.6%), post removal (2/18, 11.1%), and intraosseous anesthesia (1/18, 5.5%). Out of the 18 studies included, 12 are in vitro (66.6%), five are in vivo (case report) (27.7%), and one is ex vivo (5.5%). The DNS demonstrated accuracy and efficiency in performing minimally invasive access cavities, locating calcified canals, and performing endodontic microsurgery, and it helped target the site for intraosseous anesthesia.

## Introduction



**Video 1** Planning endodontic microsurgery in X-Guide's Implant Planning Software.


**Video 2** Dynamic navigation system (DNS) during endodontic microsurgery in real time (X-guide system).


Robotics in endodontics is no longer fiction. Inherited from implant dentistry, the dynamic navigation system (DNS) is a breakthrough technology for minimally invasive procedures. It applies a highly desired guided endodontic concept to surgical and nonsurgical procedures. The DNS is a type of tele-manipulated medical robot. 1 Tele-manipulated robots are nonautonomous master—slave robots controlled by surgeons using force-feedback haptic devices and image-guided systems. 1

**Fig. 1:**
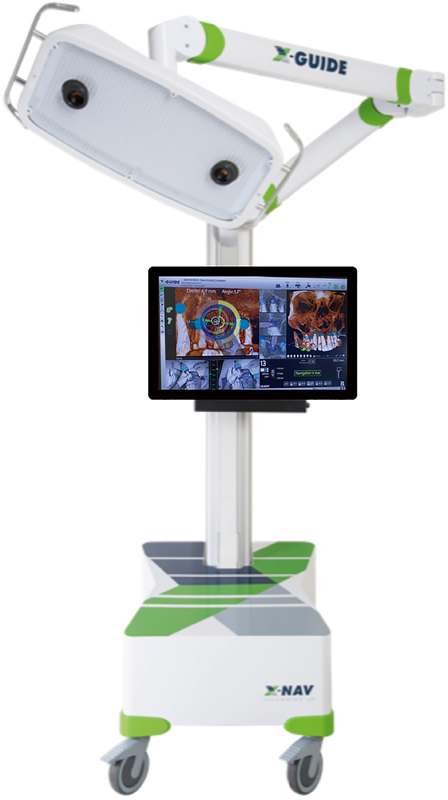
Dynamic navigation system (DNS) console.

**Fig. 2:**
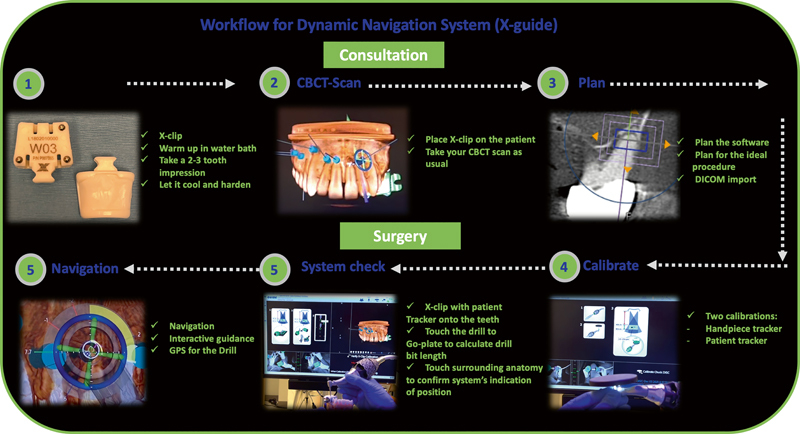
Dynamic navigation system workflow.

**Fig. 3:**
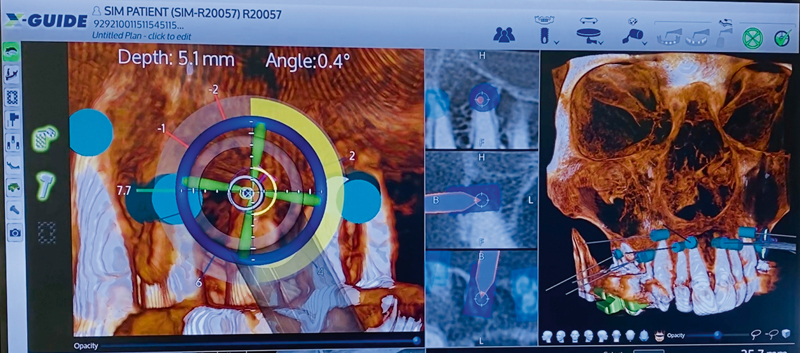
Surface of the dynamic navigation system (DNS) during endodontic microsurgery.

DNSs generally consist of a transportable workstation, a monitor, a graphic user interface with software to plan and guide therapy, and a position measuring system (a three-dimensional tracking system; Fig. 1 ). 2 The DNS is based on computer-aided surgical navigation technology and is analogous to global positioning systems or satellite navigation. The DNS workflow is simple and straightforward ( Fig. 2 ). The ideal drill position is virtually planned by the surgeon in the preoperative cone-beam computed tomography (CBCT) dataset uploaded to the planning program ( Video 1 , available in the online version only). Sensors attached to the handpiece and the patient's teeth transfer the 3D spatial information to a stereo tracker. 2 3 4 This technology has motion-tracking optical cameras and CBCT images of the position of the virtually planned surgery that provide 3D real-time dynamic navigation with visual feedback to intraoperatively guide surgical instruments ( Fig. 3 ). Most importantly, the surgeon can adjust the treatment course in real time ( Video 2 , available in the online version only).

DNS in endodontics first appeared in the literature in 2019, focusing on creating conservative access cavities and locating canals, which demonstrated its potential use in guided endodontics. 5 Since then, the DNS's potential has been explored for different applications in endodontics. Currently, the DNS has been considered for conventional and minimally invasive access cavities, 5 6 7 8 9 10 11 12 locating calcified canals, 13 14 15 16 endodontic microsurgery, 16 17 18 post removal, 19 20 and intraosseous anesthesia anesthesia. 21

The DNS is an emerging technology that can revolutionize endodontics by accurately and safely delivering minimally invasive procedures and avoiding catastrophic mishaps during complex procedures. Lately, this technology has attracted the attention of researchers and surgeons in dentistry. To help orient researchers and clinicians to future DNS applications in endodontics, we conducted this scoping review (SCR) to map the existing literature on the use of the DNS in endodontics. We examined the extent, range, and nature of research activity in this area. Additionally, this SCR disseminates research findings, determines the value of conducting a full systematic review with meta-analysis, and identifies gaps in the existing literature and directions for future research.

## Methods

### Study Design

In this SCR on using the DNS in endodontics, we adopted a five-stage framework from Arksey & O'Malley 22 and embraced Levac et al 23 recommendations. The five included framework stages are (1) identifying the research question; (2) identifying relevant studies; (3) selecting studies; (4) charting data; and (5) collating, summarizing, and reporting the results.

### Stage I: Research Question

For this study, we aimed to answer the following main question: What are the DNS applications in endodontics?

### Stage II: Identification of Pertinent Studies

With the support of a research librarian, two independent reviewers (F.C.M. and B.J.M.C.) conducted this literature research on studies published through November 2021. They conducted the electronic search using the following databases: PubMed (Medline), Scopus (Elsevier), and Web of Science (Clarivate Analytics). We used the building-block approach for the search (Concept #1: “System”; Concept #2 “Treatment modality”; and Concept #3: “Field”) with a combination of medical subject headings words and keywords ( Table 1 ). We used Boolean operators *AND* and *OR* , truncation for words with multiple endings, quotes for phrases, and nesting to group similar terms. We performed this search strategy for PubMed (Medline) and adapted it for the other selected databases. To ensure the quality assessment of discovered resources, we limited our searches to peer-reviewed journals. Additionally, we checked the references cited in the included articles to identify other potentially relevant articles.

**Table 1:** Search strategy used in PubMed.

# 1	"Apicoectomy"[Mesh] OR "Molar"[Mesh] OR "Tooth Apex"[Mesh] OR "Surgery, Oral"[Mesh] OR "Dental Cavity Preparation"[Mesh] OR "Microsurgery"[Mesh] OR "Dental Pulp Cavity"[Mesh] OR "Anesthesia, Dental"[Mesh] OR "Cone-Beam Computed Tomography"[Mesh] OR "Post and Core Technique"[Mesh] OR “Root-end resection*” [tw] OR “Root end resection*”[tw] OR “Root canal*”[tw] OR “Endodontic Access” [tw] OR “Intraosseous Anesthesia” [tw] OR “Calcified canal*”[tw] OR Access OR “Access cavit*”[tw] OR “Endodontic Retreatment*” [tw] OR “Fiber post*”[tw] OR Microsurger*[tw] OR Tooth[tw] OR “Molar surger*”[tw] OR Incisor*[tw] OR "Anesthesia, Dental"[Mesh:NoExp] OR Retreatment*[tw] OR “Root Canal-Treated”[tw]
# 2	"Surgical Navigation System*"[Mesh] OR "Robotic Surgical Procedures"[Mesh] OR “Dynamic navigation system*”[tw] OR “Dynamic navigation”[tw] OR “Dynamic technolog*”[tw] OR “Computer-aided dynamic navigation” [tw] OR “Real-time guide*” [tw] OR “3D Navigation system*”[tw] OR “Dynamic Navigation Technolog*”[tw] OR “3-Dimensional Navigation” [tw] OR “3D- Navigation system*”[tw] OR “Computer-aided navigation” [tw]
# 3	"Endodontics"[Mesh] OR "Root Canal Therapy"[Mesh] OR Endodontic* OR Endodontal[tw] OR Endodontical [tw]
# 4	#1 and #2 and #3

### Stage III: Studies Selection

We established the inclusion criteria for the studies at the beginning of the scoping process through Steps I and II. The inclusion criteria were (1) references that studied DNS in endodontics; (2) in vitro, in vivo, and ex vivo studies; (3) references in English; and (4) peer-reviewed journals. The exclusion criteria were the following: (1) references published in languages other than English; (2) articles with no interventions; (3) reviews; and (4) editorial letters. Three researchers (F.C.M., B.J.M.C., and I.L.G.) independently reviewed abstracts yielded from the search strategy for study selection. Each independent researcher decided whether the reference would be considered for full-text review. Publications not fulfilling the research selection criteria were excluded. Next, two reviewers (F.C.M. and B.J.M.C.) independently reviewed the full articles for inclusion. When disagreement occurred, a third reviewer (I.L.G.) was consulted to determine final inclusion. The search results were combined in an online management platform tool for systematic review (Covidence by Cochrane, Melbourne, Australia) Supplementary Fig. S1 (available in online version only) shows PRISMA flow diagram maps out the number of records identified, included and excluded, and the reasons for exclusions.

### Stage IV: Data Charting

We collectively developed the data-charting form to determine which variables to extract from the included studies. Afterward, we used a spreadsheet software to create a template for data extraction. The researchers were calibrated to extract and record the data. Three researchers (F.C.M., B.J.M.C., and I.L.G.) performed the data extraction in Stage IV.

### Stage V: Collating, Summarizing, and Reporting the Results

Three researchers (F.C.M., B.J.M.C., and I.L.G.) executed Stage V. The data were arranged according to (1) author, (2) year, (3) country of origin, (4) type of study (in vitro, in vivo, or ex vivo), (5) type of system (manufacturer), (6) endodontic application, (7) study design (single evaluation or comparison), and (8) main findings ( Table 2 ). The descriptive analysis captured the application of the DNS in endodontics. The following five themes emerged for DNS application in endodontic treatment: Theme 1—endodontic access cavity; Theme 2—locating calcified canals; Theme 3—endodontic microsurgery; Theme 4—post removal; and Theme 5—intraosseous anesthesia.

**Table 2:** Characteristics of the 18 included studies.

Author (Ref #)	Year	Country	Type of study	Type of system (Manufacturer)	Endodontic application	Type of study (Comparison or no comparison)
Chong et al (5)	2019	United	*In vitro*	Navident	Endodontic access cavity	DNS
		Kingdom	(Dental casts fabricated from	(ClaroNav)	(Minimally invasive)	(No comparison)
			human teeth)			
Main findings						
1. Conservative access cavities were achieved and all the expected canals were successfully located in 26 teeth.						
2. Due to tracking difficulties, only one canal was located in two maxillary second molars; in a maxillary first molar, only two canals were located and the access						
preparation for the third canal was misaligned and off-target.						
**Author (Ref #)**	**Year**	**Country**	**Type of study**	**Type of system (Manufacturer)**	**Endodontic application**	**Type of study (Comparison or no comparison)**
Zubizarreta-Macho et al (6)	2020	Spain	*In vitro*	Navident	Endodontic access cavity	Computer-aided Static (Printed guide)
			(Human extracted teeth)	(ClaroNav)	(Conventional)	*Versus*
						Computer-aided dynamic (DNS)
						*Versus*
						Manual approach
Main findings						
1. Paired t-test revealed no statistically significant differences between SN and DN at the coronal ( *p* = 0.6542) apical, ( *p* = 0.9144), or angular ( *p* = 0.0724) level.						
2. Statistically significant differences were observed between the two computer-aided navigation techniques and the MN group at the coronal ( *p* <0.0001),						
apical ( *p* <0.0001), and angular ( *p* <0.0001).						
3. Overall the DNS group (DN) were more accurate than printed guided (SN); however, they were not statistically significant.						
**Author (Ref #)**	**Year**	**Country**	**Type of study**	**Type of system (Manufacturer)**	**Endodontic application**	**Type of study (Comparison or no comparison)**
Gambarini et al (7)	2020	Italy	*In vitro*	Navident	Endodontic access cavity	Free-hand technique
			(Tooth Replica, artificial teeth)	(ClaroNav)	(Minimally invasive)	*Versus*
						Dynamic navigation system
Main findings						
1. The X1 and Y1 groups showed higher precision than the other two groups ( *p* <0.05).						
X1 = ultra-conservative access cavity planning on MB1 canal. Performed on the buccal-palatal plane (buccal view) by planning the opening axis coinciding with the						
coronal third orifice of the canal.						
Y1 = ultra-conservative access cavity planning on MB1 canal. Performed on the mesio-distal plane (mesial view) by planning the opening axis coinciding with the						
coronal third orifice of the canal.						
2. Significant differences were found between the degrees of deviations of the cavities performed hands-free and the ones performed with the DNS ( *p* <0.05).						
**Author (Ref #)**	**Year**	**Country**	**Type of study**	**Type of system (Manufacturer)**	**Endodontic application**	**Type of study (Comparison or no comparison)**
Gambarini et al (8)	2020	Italy	*In vitro*	Navident	Endodontic access cavity	Free-hand technique
			(Tooth replica, artificial teeth)	(ClaroNav)	(Minimally invasive)	*Versus*
						Dynamic navigation system
Main findings						
1. Differences were found in the tested parameters between the two groups.						
2. The DNS group was significantly more precise, showing smaller mean values in the angulation (4.8 degrees) and in the maximum distance from the ideal position (0.34 mm),						
when compared with manual approach (MA) group (mean values, 21.2 degrees and 0.88 mm, respectively).						
**Author (Ref #)**	**Year**	**Country**	**Type of study**	**Type of system (Manufacturer)**	**Endodontic application**	**Type of study (Comparison or no comparison)**
Pirani et al (9)	2020	Italy	*In vitro*	ImplaNav	Endodontic access cavity	DNS
			(Human extracted teeth)	(Navigation system)	(Minimally invasive)	(No comparison)
Main findings						
1. All access cavities were prepared according to a minimally invasive endodontics approach with the dynamically guided ImplaNav software.						
2. No perforations occurred and all the canals were successfully located.						
**Author (Ref #)**	**Year**	**Country**	**Type of study**	**Type of system (Manufacturer)**	**Endodontic application**	**Type of study (Comparison or no comparison)**
Dianat et al (10)	2021	United	*In vivo*	X-Guide system	Endodontic access cavity	DNS
		States	Case report	(X-Nav Technologies)	(Minimally invasive)	(No comparison)
			(Maxillary right first molar)			
Main findings						
1. The dynamic navigation system allowed for the successful location of the canal.						
**Author (Ref #)**	**Year**	**Country**	**Type of study**	**Type of system (Manufacturer)**	**Endodontic application**	**Type of study (Comparison or no comparison)**
Connert et al (11)	2021	Switzerland	*In vitro*	DENACAM	Endodontic access cavity	Free-hand technique
			(Human extracted teeth)	System	(Conventional)	*Versus*
						miniaturized real-time
						Guided endodontics (DNS)
Main findings						
1. Substance loss was significantly lower with real-time guided endodontics than conventional freehand method (mean = 10.5 mm ^3^ vs. 29.7 mm ^3^ ), but both						
procedures took a similar time per tooth (mean = 195 vs. 193 s).						
2. Operator 1 (more experienced) achieved significantly less substance loss than operator 2 (less experienced) with conventional freehand method (mean = 19.9 vs.						
39.4 mm ^3^ ) but not with RTGE (mean = 10.3 vs. 10.6 mm ^3^ ).						
3. Real-time guided endodontics seems to be independent of operator experience.						
**Author (Ref #)**	**Year**	**Country**	**Type of study**	**Type of system (Manufacturer)**	**Endodontic application**	**Type of study (Comparison or no comparison)**
Jain et al (12)	2020	United	*In vitro*	Navident	Endodontic access cavity	Free-hand technique
		States	(3D-printed Teeth)	(ClaroNav)	(Conventional)	*vs.*
						Dynamic navigation system
Main findings						
1. Dynamically navigated accesses resulted in significantly less mean substance loss in comparison with the freehand technique (27.2 vs. 40.7 mm ^3^ ) ( *p* <0.05).						
2. Dynamically navigated accesses were also associated with higher optimal precision (drill path centered) to locate calcified canals in comparison						
with the freehand technique (75 vs. 45%, *p* <0.05).						
3. Mandibular teeth were associated with a negligible difference in substance loss between the access techniques (19.0 vs. 19.1 mm ^3^ ) ( *p* <0.05).						
4. Qualitatively the freehand technique was still prone to 30% higher chance of suboptimal precision (drill path tangentially transported) in locating calcified canals.						
5. dynamically navigated accesses were prepared significantly faster than freehand preparations (2.2 vs. 7.06 min) ( *p* <0.05).						
**Author (Ref #)**	**Year**	**Country**	**Type of study**	**Type of system (Manufacturer)**	**Endodontic application**	**Type of study (Comparison or no comparison)**
Jain et al (13)	2020	United	*In vitro*	Navident	Locating calcified canal	DNS
		States	(In vitro 3D-printed teeth	(ClaroNav)		(No comparison)
			Surgical Jaw model)			
Main findings						
1. The mean 2D horizontal deviation from the canal orifice was 0.9 mm, and it was significantly higher on maxillary compared with mandibular teeth ( *p* <0.05).						
2. The mean 3D deviation from the canal orifice was 1.3 mm, and it was marginally higher on maxillary teeth in comparison with mandibular teeth ( *p* <0.05).						
3. The mean 3D angular deviation was 1.7 degrees, and it was higher in molars compared with premolars ( *p* <0.05).						
4. The 3D and 2D discrepancies were independent of the canal orifice depths ( *p* <0.05).						
5. The mean 2D horizontal deviation from the canal orifice was 0.9 mm, and it was higher on maxillary compared with mandibular teeth ( *p* <0.05).						
6. The average drilling time was 57.8 s with significant dependence on the canal orifice depth, tooth type, and jaw ( *p* <0.05).						
**Author (Ref #)**	**Year**	**Country**	**Type of study**	**Type of system (Manufacturer)**	**Endodontic application**	**Type of study (Comparison or no comparison)**
Dianat et al (14)	2020	United	*In vitro*	X-Guide system	Locating calcified canal	Free-hand technique
		States	(Human extracted teeth)	(X-Nav Technologies)		*vs.*
						Dynamic navigation system
Main findings						
1. The mean linear and angular deviations, reduced dentin thickness (at both levels), the time for access cavity preparation and the number of mishaps in the DNS						
group were significantly less than the FH group ( *p* <0.05).						
2. The unsuccessful attempts were not different between the two groups ( *p* <0.05).						
3. The time for access preparation was significantly shorter for the board-certified endodontist in the FH group ( *p* <0.05).						
**Author (Ref #)**	**Year**	**Country**	**Type of study**	**Type of system (Manufacturer)**	**Endodontic application**	**Type of study (Comparison or no comparison)**
Torres et al (15)	2021	Belgium	*In vitro*	Navident	Locating calcified Canal	DNS
			(3D-printed teeth)	(ClaroNav)		(No comparison)
Main findings						
1. All operators located a total of 156 canals, obtaining an overall success of 93% without a difference between operator experience ( *p* >0.05).						
2. The mean deviation at the apical point was 0.63 mm (SD 0.35 mm) and was significantly lower in anterior teeth in comparison with molars ( *p* <0.05).						
3. The mean angular deviation from the planning was 2.81 degrees (SD 1.53 degrees).						
**Author (Ref #)**	**Year**	**Country**	**Type of study**	**Type of system (Manufacturer)**	**Endodontic application**	**Type of study (Comparison or no comparison)**
Dhesi and Chong (16)	2020	United	*In vivo*	Navident	Locating calcified canal	DNS
		Kingdom	Case report	(ClaroNav)		(No comparison)
			(Maxillary right second premolar)			
Main findings						
1. The DNS allowed to safe and accurate location and negotiation of obliterated and narrowed canals faintly visible and pulp space obliterated.						
**Author (Ref #)**	**Year**	**Country**	**Type of study**	**Type of system (Manufacturer)**	**Endodontic application**	**Type of study (Comparison or no comparison)**
Gambarini et al (17)	2019	Italy	*In vivo*	Navident	Endodontic microsurgery	DNS
			Case report	(ClaroNav)		(No comparison)
			(Maxillary right lateral incisor)			
Main findings						
1. The system allowed precise localization of the root and precise apicoectomy with a minimal invasive cavity.						
2. The dynamic navigation system allowed the student to precisely direct the bur in 3 dimensions.						
3. The osteotomy and root-end resection were easily and quickly performed by an undergraduate student with a minimally invasive approach without iatrogenic errors.						
4. The navigation system allowed the operator to precisely perform a minimally invasive osteotomy and root-end resection during endodontic surgery.						
**Author (Ref #)**	**Year**	**Country**	**Type of study**	**Type of system (Manufacturer)**	**Endodontic application**	**Type of study (Comparison or no comparison)**
Dianat et al (18)	2021	United	*Ex vivo*	X-Guide system	Endodontic microsurgery	Free-hand technique
		States	(Human teeth in fresh	(X-Nav Technologies)		*Versus*
			cadaver head)			Dynamic navigation system
Main findings						
1. Linear deviations, angular deflection and operation time were significantly less in the DNS group ( *p* <0.05).						
2. The number of mishaps was not different between the two groups ( *p* >0.05).						
3. Sub- group analyses revealed that the distance of >5 mm from buccal cortical plate was significantly associated with lower accuracy, increased operation time						
greater incidence of mishaps in the FH group ( *p* <0.05), but not in the DNS group.						
**Author (Ref #)**	**Year**	**Country**	**Type of study**	**Type of system (Manufacturer)**	**Endodontic application**	**Type of study (Comparison or no comparison)**
Lu et al (26)	2022	Taiwan	*In vivo*	X-Guide system	Endodontic microsurgery	DNS
			Case report	(X-Nav Technologies)		(No comparison)
			(Mandibular left second molar)			
Main findings						
1. Endodontic microsurgery with the aid of dynamic navigation, especially in anatomically challenging scenarios, is a promising procedure.						
2. The intactly removed buccal cortical plate by a navigated trephine bur can be served as autograft to enhance post-operative healing.						
**Author (Ref #)**	**Year**	**Country**	**Type of study**	**Type of system (Manufacturer)**	**Endodontic application**	**Type of study (Comparison or no comparison)**
Bardales-Alcocer et al (19)	2021	Canada	*In vivo*	Navident	Root post removal	DNS
			Case report	(ClaroNav)		(No comparison)
			(Maxillary left lateral incisor)			
Main findings						
1. The dynamic navigation system enabled minimally invasive removal of the fiber post with a high degree of accuracy, thus ensuring that there was no unnecessary						
removal of root structure.						
2. Dynamic navigation using real-time monitoring could reduce the attendant risk of iatrogenic errors in complex treatment cases.						
**Author (Ref #)**	**Year**	**Country**	**Type of study**	**Type of system (Manufacturer)**	**Endodontic application**	**Type of study (Comparison or no comparison)**
Janabi et al (20)	2021	United	*In vitro*	X-Guide system	Root post removal	Free-hand Technique
		States	(Human extracted teeth fixed in	(X-Nav Technologies)		*Versus*
			tissue-denied cadaver maxilla)			Dynamic Navigation system
Main findings						
1. The DNS group showed significantly less global coronal and apical deviations and angular deflection than the FH group ( *p* <0.05).						
2. DNS required less operation time than FH.						
3. the DNS technique had significantly less volumetric loss of tooth structure than the FH technique ( *p* < 0.05).						
**Author (Ref #)**	**Year**	**Country**	**Type of study**	**Type of system (Manufacturer)**	**Endodontic application**	**Type of study (Comparison or no comparison)**
Jain et al (21)	2020	United	*In vitro*	Navident	Intraosseous	Free-hand Technique
		States	(In vitro 3D-printed teeth	(ClaroNav)	Anesthesia	*Versus*
			Surgical Jaw model)			Dynamic Navigation system
Main findings						
1. The rate for perforation was significantly higher for the FH group than the dynamic navigation ( *p* <0.05).						
2. For dynamic navigation, the 2D entry deviation was 0.71 mm (95% confidence interval [CI], 0.56–0.87).						
3. The mean 2D horizontal deviation was 0.96 mm (95% CI, 0.79–1.14), and the mean 2D vertical deviation was 0.70 mm (95% CI, 0.55–0.84).						
4. The 3D deviation at the tip was on an average 1.23 mm (95% CI, 1.05–1.42).						
5. The overall 3D angular deviation was on average 1.36° (95% CI, 1.15–1.56).						
6. The inter-radicular distance was not significantly associated with any 2D or 3D discrepancies.						

## Results

### Characteristics and the Type of Included Studies

**Fig. 4:**
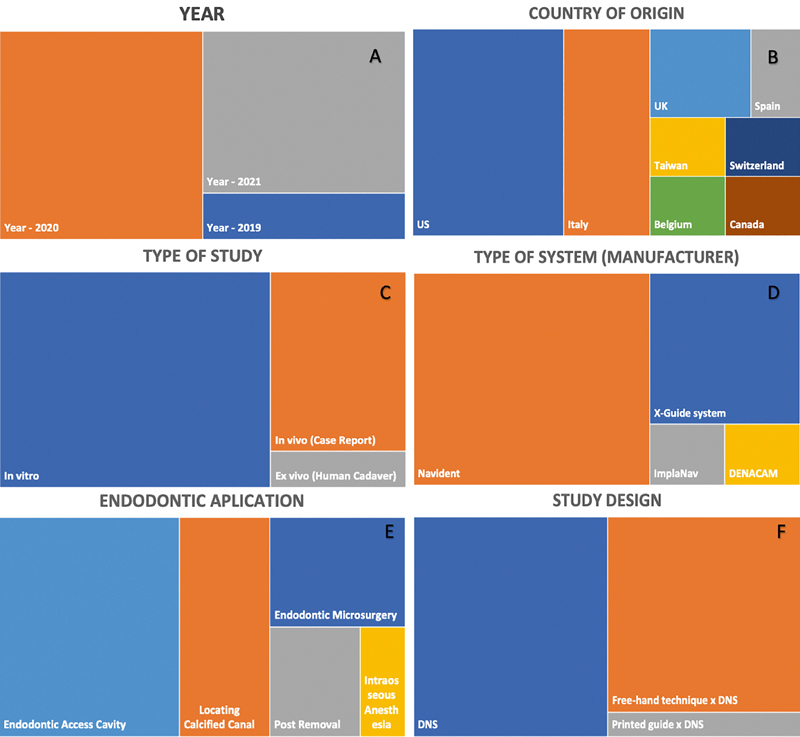
Characteristics of included studies: **(A)** year of publication; **(B)** country of origin; **(C)** type of study; **(D)** type of system (manufacturer); **(E)** endodontic application; and **(F)** study design.

Table 2 shows the characteristics of the studies, outcomes, and main findings included here. The use of DNS in endodontics was recently explored with articles published from 2019 to 2021 ( Fig. 4A ). Most of the studies were conducted in the United States (7/18, 38.8%), Italy (4/18, 22.2%), and the United Kingdom (2/18, 11.11%), followed by Taiwan, Spain, Belgium, Switzerland, and Canada (1/18, 5.5% each; Fig. 4B ). Of the 18 included studies, 12 were in vitro (66.6%), five were in vivo (case reports; 27.7%), and one was ex vivo (a human cadaver study; 5.5%; Fig. 4C ). Four different DNS manufacturers were evaluated in these studies: Navident (11/18, 61.1%), the X-Guide system (5/18, 27.7%), ImplaNav (1/18, 5.5%), and the DENACAM system (1/18, 5.5%; Fig. 4D ). The DNS was explored for different endodontic applications, including access cavity preparation (8/18, 44.4%), calcified canal location (4/18, 22.2%), microsurgery (3/18, 16.6%), post removal (2/18, 11.1%), and intraosseous anesthesia (1/18, 5.5%; Fig. 4E ). Nine studies (9/18, 50%) were single-evaluation (only DNS was evaluated), eight studies (8/18, 44.4%) compared free hand (FH) and DNS, and one study (1/18, 5.5%) compared printed guide (computer-aided static technique), DNS (computer-aided dynamic technique), and FH ( Fig. 4F ).

### Themes: Current Applications of DNS in Endodontics

#### Theme 1: Endodontic Access Cavity

*Minimally invasive endodontic access cavity:* Lately, minimally invasive endodontics (MIE) has been debated. 5 7 8 9 The idea behind MIE is performing endodontic treatment with minimal loss of tooth structure, aiming for high tooth preservation. However, there are some cases in which MIE is difficult to achieve with the FH technique. The DNS has been evaluated for MIE. 5 7 8 9 Chong et al 5 in an in vitro study, successfully performed conservative access cavity in dental casts fabricated from sets of extracted teeth. The DNS was successfully used despite tracking difficulties in some molars. Gambarini et al 7 described and classified four different types of point endodontic access cavities (PEACs). The authors verified in vitro that DNS allowed planning and precise execution of these cavities in artificial resin upper right first molars. The DNS allowed for minimally invasive preparation with some differences across the PEACs. The same researchers 8 showed in vitro the benefit of DNS in performing ultraconservative access cavities in resin upper right first molars. The DNS minimized the potential risk of iatrogenic weakening of critical portions of the crown and reduced negative influences on shaping procedures. Pirani et al 9 taught the in vitro application of DNS to undergraduate students for performing MIE in extracted human teeth, and all MIE access cavities were completed without mishaps.

*Conventional endodontic access cavity:* Different studies have shown that the long-term survival of root-canal-treated teeth is often associated with major restorations. 24 25 Therefore, saving tooth structure when performing conventional access cavities is critical. The DNS has been evaluated for conventional endodontic access cavities. 6 10 11 12 Zubizarreta-Macho et al 6 compared in vitro the accuracy of computer-aided dynamic (DNS), computer-aided static (printed guide), and FH methods to prepare endodontic access cavities in single-rooted anterior teeth. The authors revealed no difference between the DNS and the printed guide at the coronal, apical, or angular levels, with both exhibiting higher accuracy than FH. Dianat et al 10 in a case report, located the distobuccal canal partially calcified on a maxillary right first molar with a narrow pulp chamber. Connert et al 11 evaluated in vitro substance loss and the time required for access cavity preparation. They used a miniaturized DNS of real-time guided endodontics (RTGE) and conventional FH (CONV) in human anterior maxillary teeth between two dentists with 2 and 12 years of endodontic experience. Overall, the substance loss was lower for the RTGE than for the CONV, with both procedures lasting for the same amount of time. The more experienced operator achieved less substance loss than the operator with less experience with CONV but not with RTGE. This proved that RTGE's effectiveness is independent of operator experience. Jain et al 12 compared in vitro DNS's and FH's speeds, qualitative precisions, and quantitative losses of tooth structure in 3D-printed maxillary and mandibular central incisors. The DNS resulted in less substance loss, higher optimal precision in locating calcified canals, and faster access preparation than the FH.

#### Theme 2: Locating Calcified Canal

Access to calcified or obliterated root canals can be challenging and time-consuming for even the most experienced endodontists. Indistinct canal paths or canals not visible on a radiograph entail an increased risk of mishaps, such as excessive dentin removal and perforation. Previous studies have explored the DNS's potential to locate calcified canals. 13 14 15 16 Jain et al 13 evaluated in vitro the accuracy of the DNS in locating complex simulated canals in three identical sets of maxillary and mandibular teeth. The mean 2D horizontal deviation from the canal orifice was higher on maxillary teeth than on mandibular teeth. The 3D angular deviation was higher in premolars than molars, with the average drilling time dependent on the canal orifice depth, tooth type, and jaw. Dianat et al 14 compared the accuracy and efficiency of DNS and FH in locating calcified canals in single-rooted teeth with canal obliteration mounted in dry cadaver jaws. The mean linear and angular deviations, reduced dentin thickness, the time for access preparation, and the number of mishaps were significantly less frequent with the DNS than with the FH. In a case report with the adjunct of the DNS, Dhesi and Chong 16 located and accessed the canal in a maxillary second premolar with the pulp space completely obliterated and the narrowed canals faintly visible. More recently, Torres et al 15 evaluated the in vitro accuracy of DNS. Three operators with different training levels prepared access cavities in teeth with severe pulp canal obliteration in 3D-printed jaws. The three operators achieved an overall success rate of 93%, regardless of the operator's experience.

#### Theme 3: Endodontic Microsurgery

Endodontic microsurgery can predictably address persistent or recurrent apical periodontitis associated with root canal treatment. However, osteotomy and root-end resection can be challenging in several circumstances. Obtaining surgical access to mandibular molars with apices far from the buccal cortical bone is difficult. Important anatomical structures such as the maxillary sinus, mental foramen, and mandibular canal are also concerns during surgery. Additionally, surgical time is a critical factor for endodontic microsurgeries. Clinicians prefer shorter surgical procedures to avoid operator and patient fatigue, loss of anesthesia, and excessive bleeding, which can compromise visibility and ultimately the procedure's outcome. Some endodontists avoid endodontic microsurgeries because of the difficulties in such procedures. Hence, new technologies such as DNS are needed to facilitate more accurate and efficient surgical access of root apices. 17 18 26 Gambarini et al, 17 in a case report, covered an undergraduate student's use of DNS for osteotomy and root-end resection in symptomatic upper lateral incisor with persistent apical periodontitis. The DNS system allowed the student to perform a minimally invasive osteotomy and a precise root-end resection. The authors suggested that the DNS could facilitate the operator's maneuvers and reduce the risk of errors. More recently, Dianat et al 18 compared the accuracy and efficiency of the DNS to FH, CBCT scan, and a dental operating microscope (DOM). The authors conducted root-end resection in 40 roots in cadaver heads. The DNS was more accurate and efficient in root-end resection with significantly less global deviation (platform and apex) and angular deflection, and it required less time than FH. However, the distance from the roots to the cortical plate negatively affected the DNS's accuracy and efficiency. Moreover, the DNS and FH showed no difference in mishaps. In a case report, Lu et al 26 used the DNS in endodontic microsurgery in a mandibular left second molar of a patient with intermittent pain and a sinus tract. The DNS allowed an accurate localization of the root tip and decreased the preparation time. Moreover, it aided achievement of an ideal root-end resection with no bevel.

#### Theme 4: Post Removal

Removing posts from endodontically treated teeth is frequently necessary in cases of root canal failure. Post removal is challenging because of risks such as deviating from the root apex, unnecessary removal of sound root dentin, micro-cracks, and root fracture. 27 28 Given these challenges, managing persistent or recurrent apical periodontitis appears to be a perplexing dilemma, and decisions regarding its treatment vary among clinicians. 29 There are multiple post-removal systems and techniques described in the literature. Although the FH technique of drilling out the post with dental burs or ultrasonic tips is the most common, this technique has multiple disadvantages. It is time-consuming and requires removing a significant coronal tooth structure to visualize the post under the DOM. 30 Moreover, determining the post's angulation and establishing the drilling path demand significant clinical experience. One of the advantages of the DNS is a real-time visualization of the position and the drill's angulation, which allows alteration of the plan during the procedure if needed. Bardales-Alcocer et al, 19 in a case report, performed post removal during nonsurgical retreatment in a maxillary lateral incisor supporting a zirconium bridge extending from Teeth 8 and 10 guided with the DNS. The DNS enabled minimally invasive removal of the fiber post with high accuracy. The authors suggested the DNS could reduce the risk of iatrogenic errors. Janabi et al 20 recently investigated the accuracy and efficiency of the DNS compared with FH. They removed fiber posts from endodontically treated human maxillary teeth mounted in a tissue-denuded cadaver maxilla. The DNS showed less coronal and apical deviations and angular deflection than the FH. Overall, the FH technique required twice as much time (8.30 ± 4.65 minutes) as the DNS (4.03 ± 0.43 minutes). Furthermore, the DNS resulted in significantly less volumetric (mm ^3^ ) tooth structure loss than FH.

#### Theme 5: Intraosseous Anesthesia

Profound anesthesia can be critical for pain control on a patient diagnosed with symptomatic irreversible pulpitis (also known as a *hot tooth* ). Some studies using varying local anesthesia protocols with different anesthetics and supplemental techniques have had low success. 31 32 Intraosseous anesthesia is a supplemental technique with a predictable success rate of over 70%. 32 33 34 Despite its high success rate, the drill tip's precise orientation can be challenging. It may influence the endodontist to choose a less effective supplemental technique, such as PDL ligament injection. 35 Recently, Jain et al 21 compared in vitro the accuracy and efficiency of the DNS to those of FH in delivering intraosseous anesthesia in 3D print surgical models. The rate of root perforation was higher for the FH, and there was no perforation with the DNS. The 2D entry, horizontal deviation, and 3D deviation of the tip for the DNS resulted in accurate drilling at 100% of the injection sites.

## Discussion

Most studies were in vitro using models such as extracted human teeth, 3D-printed teeth, tooth replicas, surgical jaw models, and extracted human teeth fixed in tissue-denuded cadaver maxilla. 5 6 7 8 9 11 12 13 15 20 21 Of the studies included here, only 27% of the studies were in vivo, but all were case reports. 10 16 17 19 26 In these case reports, five patients were treated with the DNS approach. Microsurgery studies involved a maxillary right lateral incisor 17 and a mandibular left second molar, 26 two studies focused on locating calcified canals (one in a maxillary right second premolar 16 and the other in a maxillary right first molar 10 ), and one study covered post removal in a maxillary left lateral incisor. 19 One study was ex vivo, conducted in human teeth in a fresh cadaver head for endodontic microsurgery. 18

Most of the previous studies included here were single evaluations of the DNS. 5 9 10 15 16 17 19 21 26 The majority of the comparison studies compared DNS and the FH technique, 7 8 11 12 13 14 18 20 in which the robot's accuracy and precision are expected to be higher than a human surgeon. Reconciling the data from comparison studies involving the FH technique can be critical, mainly because the surgeon's training and hand skills could be confounding factors. It is worth pointing out that although the DNS is a computer-aided navigation approach, the surgeon manually operates the handpiece. Small hand tremors can be captured by the DNS camera. Whether endodontic training and hand skills influence DNS accuracy and precision is debated. However, most studies indicate that the accuracy and precision of the DNS are independent of the operator's skills, 11 14 17 which makes DNS a valuable tool for teaching undergraduate students. 9 Although the DNS technique has a learning curve, in general, 20 trial attempts are necessary for learning and calibration before patient intervention seems to be adequate according to previous investigations. 13 14 18 20 The DNS technique also requires certain hand–eye coordination. Manual dexterity must be continuously maintained by the operator throughout the procedure while they look at the computer screen. Currently, there is only one comparison study of DNS (computer-aided dynamic technique) versus the computer-aided static approach (printed guide) for endodontic access cavities. 6 The authors reported no statistically significant difference between the two computer-aided techniques for most accuracy metrics. It should be noted that these findings must be interpreted with caution because this study has no sample size calculation.

Here, we identified four DNS technologies used for endodontic procedures. These technologies include Navident (ClaroNav), the X-Guide system (X-Nav technologies), ImplaNav (Navigation system), and the DENACAM system. Although all four DNS technologies apply the same principle of real-time navigation, each of them has inherent advantages and disadvantages. At this time, there is no study comparing the accuracy of different DNS technologies.

Up to now the endodontic procedures have been planned under the implant software with the tools that are available ( Supplementary Fig. S2 , available in online version only). Therefore, the accuracy metrics were inherited from implant dentistry. The DNS accuracy for implant delivery can be determined by superimposing the preoperative virtual surgical plan and the postoperative CBCT scan ( Supplementary Fig. S3 , available in online version only). Then, software is used to quantify deviations of the delivered implant from the planned position and orientation. Because of the limited number of in vitro studies and complete absence of randomized clinical trials (RCTs), there is insufficient evidence to establish DNS accuracy values or safety range values for endodontic procedures. However, it is reasonable to assume lower deviation values from the preoperative CBCT ideal are more accurate. Table 3 shows a summary of accuracy metrics found across the DNS endodontic studies included here. It is worth pointing out that standardized terminology and measurement types are essential for the correct understanding and comparability of accuracy across reports. This SCR verified that the metrics adopted for DNS accuracy across endodontic studies are similar although sometimes named differently.

**Table 3:** Accuracy metrics of the 18 included studies.

Author	Application	Main metrics						
Chong et al (2019) (5)	Endodontic access cavity							
	(Minimally invasive)	Conservative access cavity was achieved and all the expected canals were located in 26/29 teeth						
Zubizarreta-Macho et al (2020) (6)	Endodontic access cavity	Coronal		Mean	SD	Minimum	Maximum	*p* -Value
	(Conventional access)		SN	7.44	1.57	5.40	10.00	SN-DN = 0.654
			SD	3.14	0.86	2.00	5.10	SN-MN <0.001
			MN	4.03	1.93	1.10	7.10	DN-MN <0.001
		Apical	SN	7.13	1.73	4.80	9.80	SN-DN = 0.914
			SD	2.48	0.94	1.10	3.80	SN-MN <0.001
			MN	2.43	1.23	0.80	4.50	DN-MN <0.001
		Angular	SN	10.04	5.2	4.10	19.40	SN-DN = 0.072
			SD	5.58	3.23	1.70	10.40	SN-MN <0.001
			MN	14.95	11.15	0.80	29.70	DN-MN <0.001
Gambarini et al (2020) (7)	Endodontic access cavity	Group	Angular deviation (degree)					
	(Minimally invasive)	X1	3.6 ± 0.4					
		X2	3.4 ± 0.3					
		Y1	7.1 ± 0.8					
		Y2	7.2 ± 0.7					
Gambarini et al (2020) (8)	Endodontic access cavity		Angulation (0)		Maximum distance (mm)		Time (seconds)	
	(Minimally invasive)	MA	19.2 (±8.6) ( *p* <0.05)		0.88 (±0.41) ( *p* <0.05)		12.2 (±3.2)	
		DNS	4.8 (±1.8) ( *p* <0.05)		0.34 (±0.19) ( *p* <0.05)		11.5 (±2.4)	
Pirani et al (2020) (9)	Endodontic access cavity							
	(Minimally invasive)	No perforation occurred and all canals located						
Dianat et al (2021) (10)	Endodontic access cavity	Case report (No accuracy metrics)						
	(Minimally invasive)							
Connert et al (2021) (11)	Endodontic access cavity		Operator 1		Operator 2		Median	
	(Conventional access)	Substance Loss (mm ^3^ ) – RTGE	10.3 (6.4-14.2) ( *p* = 0.008)		10.6 (6.0-15.2) ( *p* <0.001)		10.5 (7.6-13.3) ( *p* <0.001)	
		Substance Loss (mm ^3^ ) – Conv	19.9 (13.9-25.9)		39.4 (32.4-46.4)		29.7 (24.2-35.2)	
		Procedure Time (s) – RTGE	90 (62-118) ( *p* = 0.057)		305 (209-402)( *p* = 0.392)		195 (135-254) ( *p* = 0.955)	
		Procedure Time (s) – Conv	124 (100-150)		265 (242-288)		193 (164-222)	
Jain et al (2020) 21	Endodontic access cavity		Total substance loss (95% CI) mm ^3^ ) ( *p* = 0.0001)		Treatment duration (s) (95% CI) ( *p* = 0.0206)			
	(Conventional access)		Freehand	Dynamic navigation	Freehand		Dynamic navigation	
		Maxilla	62.2 (56.0-38.3)	35.5 (29.3-41.7)*	598.8 (370.0-82.6)		164.8 (101.1-228.4)*	
		Mandible	19.1 (13.0-25.3)	19.0 (12.8-25.2)	250.8 (190.6-311.0)		107.5 (76.6-138.4)*	
		Mean	40.7 (29.1-52.2)	27.2 (22.0-32.5)*	424.8 (289.4-560.2)		136.1 (101.4-170.8)*	
Jain et al (2020) (13)		(Mean, ± SD)		Jaw		Tooth Type		
	Locating calcified canal		Overall	Maxilla	Mandible	Anterior	Premolar	Molar
		Total time (s)	57.8 ± 61.91	45.6 ± 41.2	67.2 ± 72.89	142.1 ± 63.46	18.2 ± 8.11	32.2 ± 21.14
		Canal orifice depth (mm)	12.4 ± 4.04	13.6 ± 3.71	11.5 ± 4.08	18.8 ± 1.83	10.2 ± 1.84	10.2 ± 0.89
		2D Deviation - entry (mm)	1.1 ± 0.80	0.9 ± 0.65	1.2 ± 0.87	1.0 ± 0.80	1.2 ± 0.82	1.0 ± 0.80
		2D horizontal - canal orifice (mm)	0.9 ± 0.69	1.0 ± 0.78	0.7 ± 0.51	0.80 ± 0.57	0.8 ± 0.60	0.9 ± 0.77
		2D vertical - canal orifice (mm)	1.0 ± 0.64	0.9 ± 0.68	1.0 ± 0.60	0.9 ± 0.63	0.7 ± 0.52	1.1 ± 0.66
		3D Deviation - canal (mm)	1.3 ± 0.65	1.2 ± 0.57	1.4 ± 0.70	1.3 ± 0.59	1.1 ± 0.56	1.4 ± 0.71
		3D angular deviation - Canal orifice (o)	1.7 ± 0.98	1.7 ± 0.90	1.7 ± 1.04	1.5 ± 0.78	1.4 ± 0.62	1.9 ± 1.14
Dianat et al (2020) (14)	Locating calcified canal	Linear deviation (mm)						
		BL	0.19 ± 0.21 ( *p* ≤0.001)					
		MD	0.12 ± 0.14 ( *p* >0.05)					
		Angular deflection (o)	2.39 ± 0.85 ( *p* ≤0.0001)					
		CEJ (mm)	1.06 ± 0.18 ( *p* ≤0.0001)					
		End drilling point (mm)	1.18 ± 0.17 ( *p* ≤0.001)					
		Calcification category						
		9–13 mm	>13 mm	Minimum depth	Maximum depth	Calcification depth	Maxillary teeth	Mandibular teeth
	DNS (O.D.)	8	7	10.9	20	13.22 ± 2.14	6	9
	DNS (A.N.)	9	6	9.5	14.6	11.96 ± 1.52	6	9
	DNS, Total	17	13	9.5	20	12.59 ± 1.93	12	18
	FH (O.D.)	9	6	9.1	14.9	11.44 ± 1.57	6	9
	FH (A.N.	9	6	9.1	15.1	12.06 ± 1.70	7	8
	FH, Total	18	12	9. 1	15.1	11.75 ± 1.65	13	17
		Time required for access cavity, frequency of successful attempts and mishaps						
		Mean time	Minimum time	Maximum time	Successful attempts		Perforation	Gouging
	DNS (O.D.)	244 ± 1,112 s (4',4")	148 s (2', 28")	148 s (2'28")	14/15		0	1
	DNS (A.N.)	210 ± 80 s (3'30")	91 s (1', 31")	360 s (6')	15/15		0	0
	DNS, Total	227 ± 97 s (3'47")	91 s	600 s	29/30		0	1
	FH (O.D.)	568 ± 248 s (9' 28")	240 s (4')	1140 s (19')	13/15		2	2
	FH (A.N.	242 ± 83 s (4''2")	84 s (1', 24")	364 s (6', 4")	12/15		3	1
	FH, Total	405 ± 246 s (6'45")	84 s	1140 s	25/30		5	3
Torres et al (2021) ^21^	Locating calcified canal		Mean	Median	SD	Minimum	Maximum	
		Deviation at entry (mm)	0.67	0.60	0.34	0.02	1.85	
		Apical deviation (mm)	0.63	0.58	0.35	0.07	1.86	
		Vertical deviation	1.37	1.08	1.01	0.01	5.12	
		Angular deviation (o)	2.81	2.60	1.53	0.20	9.42	
		Total deviation	1.60	1.36	0.95	0.22	5.28	
		Length (mm)	14.53	15.15	1.81	9.59	17.51	
		Volume (mm ^3^ )	20.95	19.28	7.13	8.23	54.79	
Dhesi and Chong (2020) (16)	Locating calcified canal	Case report (No accuracy metrics)						
Gambarini et al (2019) (17)	Endodontic microsurgery	Case report (No accuracy metrics)						
Dianat et al (2021) (18)	Endodontic	Accuracy measures	DNS		FH		*p* -Value	
	Microsurgery	Linear deviation						
		Global platform (mm)	0.7 ± 0.19 ≤5 mm:0.73 ± 0.38 mm		2.25 ± 1.28 mm		≤0.0001	
			>5 mm: 0.68 ± 0.49 mm		≤5 mm: 1.53 ± 0.74 mm			
					>5 mm: 3.07 ± 0.78 mm			
		*p* -Value	NS		<0.001			
		Global apex (mm)	0.65 ± 0.09 mm		1.71 ± 0.51 mm		<0.0001	
			≤5 mm: 0.63 ± 0.33 mm		≤5 mm: 1.36 ± 0.39 mm			
			>5 mm: 0.65 ± 0.27 mm		>5 mm: 2.09 ± 0.86 mm			
		*p* -Value	NS		<0.001		≤0.0001	
		Angular deflection (o)	2.54 ± 2.62		12.38 ± 13.01			
			≤5 mm: 2.7 ± 2.1		≤5 mm: 10.85 ± 3.72			
			>5 mm: 2.44 ± 0.97		>5 mm: 14.54 ± 2.73			
		*p* -Value	NS		0.02			
Lu et al (2022) (26)	Endodontic microsurgery	Case report (No accuracy metrics)						
Bardales-Alcocer et al (2021) (19)	Endodontic microsurgery	Case report (No accuracy metrics)						
Janabi et al (2021) (20)	Post removal	Measurement	DNS		FH		*p* -Value	
		Global coronal deviation (mm)	0.91 ± 0.65		1.13 ± 0.84		<0.05	
		Global apical deviation (mm)	1.17 ± 0.64		1.68 ± 0.85		<0.05	
		Angular deflection (o)	1.75 ± 0.63		4.49 ± 2.10		<0.05	
		Operation time (min)	4.03 ± 0.43		8.30 ± 4.65		<0.05	
		Volume of tooth structure (mm ^3^ )	Before = 542.50 ± 81.97		Before = 571.34 ± 132.05		<0.05	
			After = 487.87 ± 74.70		After = 533.16 ± 133.12		<0.05	
Jain et al (2020) (22)	Intraosseous	Inter-radicular distance (mm)	2D horizontal tip (mm)	2D vertical tip (mm)	3D deviation tip (mm)	2D deviation entry (mm)	3D angular deviation (degree)	
	Anesthesia		*p* = 0.2183	*p* = 0.1989	*p* = 0.0926	*p* = 0.4408	*p* = 0.2145	
		1.5–2.5	0.78 ± 0.14	0.53 ± 0.12	0.99 ± 0.14	0.6 ± 0.12	1.18 ± 0.16	
		2.5–3.5	1.13 ± 0.14	0.83 ± 0.12	1.44 ± 0.14	0.71 ± 0.12	1.32 ± 0.16	
		3.5–4.5	0.97 ± 0.14	0.72 ± 0.12	1.27 ± 0.14	0.83 ± 0.12	1.57 ± 0.16	
		Overall	0.96 ± 0.09	0.70 ± 0.12	1.23 ± 0.09	0.71 ± 0.07	1.36 ± 0.10	

Abbreviations: AN, Ali Nosrat; Conv, conventional freehand method; DNS, dynamic navigation system; FH, freehand; MA, manual approach; MN, manual (freehand); NS, not significant; OD, Omid Dianat; Operator 1, 12 y of professional experience in the field of endodontics; Operator 2, 2 y of professional experience in the field of endodontics; RTGE, real-time guided endodontics; SD, computer-aided dynamic navigation system; SN, computer-aided static navigation system; X1, ultra-conservative access planning on MB1 canal. Performed on the buccal-palatal plane (buccal view) by planning the opening axis coinciding with the coronal third orifice of the canal; X2, ultra-conservative access cavity planning, on MB1 canal. Performed on the buccal-palatal plane (buccal view) by planning a straight-line access following the axis of the median-apical part of the canal; Y1, ultra-conservative access cavity planning on MB1 canal. Performed on the mesio-distal plane (mesial view) by planning the opening axis coinciding with the coronal third of the canal; Y2, ultra-conservative access cavity planning on MB1 canal. Performed on the mesio-distal plane (mesial view) by planning a straight-line access following the axis of the axis of the median-apical part of the canal.

Source: Adapted from Zubizarreta-Macho et al 2020 ^6^ ; Gambarini et al 2020 ^7^ ; Gambarini et al 2020 ^8^ ; Connert et al 2021 ^11^ ; Dianat et al 2020 ^14^ ; Dianat et al 2021. ^18^

Several advantages and limitations of the DNS are described across the included studies. However, before the DNS becomes a reality for future endodontics, certain modifications are needed. The bulky handpiece tracker attachment makes the DNS uncomfortable for routine endodontic use ( Supplementary Fig. S4 , available in online version only). Printing the DNS tracker references directly on the body of the handpiece would eliminate the need for the tracker attachment. Another option would be to create a smaller and lighter handpiece tracker device that would be easier to grip. Third, although using indirect vision to look at the display during the DNS procedure is ergonomic, it is hard to avoid losing track of the operation/treatment field. The application of augmented reality devices and head-mounted displays could be helpful.

Overall, the DNS workflow is simple and straightforward, and it easily relates to existing procedures. First, the stability of the fiducial for scan, the quality of the CBCT scan, and the preplanning accuracy are critical elements of the DNS technique. Collectively, the included DNS studies suggest that the DNS is a promising tool for different endodontic procedures. The DNS can accurately and safely deliver minimally invasive procedures. Moreover, the DNS can save procedure time in complex cases involving location of calcified canals, post removal, and endodontic microsurgery in areas that are difficult to access or visualize.

This SCR did not obtain the full value of conducting a full systematic review with meta-analysis to establish DNS accuracy values or safety range values for endodontic procedures. The number of DNS studies in endodontics is limited. Particularly, there is a lack of clinical studies and no RCTs. To help determine the DNS accuracy for endodontic procedures, future clinical studies and RCTs indicating the clinical accuracy metrics values are important. Studies are needed to challenge the DNS's accuracy in areas of access or visualization difficulty and those where there are chances of damaging important anatomical structures. Additionally, more studies are needed to compare the accuracy of the computer-aided dynamic technique (DNS) with that of the computer-aided static method (printed guide) and those of other computer-aided technologies.

## Conclusion

The DNS demonstrated accuracy and efficiency in performing minimally invasive access cavities, locating calcified canals, and performing endodontic microsurgery, and it helped target the site for intraosseous anesthesia.
