# Supplementary material for: Current Applications of Dynamic Navigation System in Endodontics: A Scoping Review
Source: Eur J Dent. 2022 Aug 31;17(3):569–86. doi: 10.1055/s-0042-1749361 (PMC10569848; doi:10.1055/s-0042-1749361)
Supplement: Supplementary file 3 — Supplementary Material [file 10-1055-s-0042-1749361-s2232016.pdf]

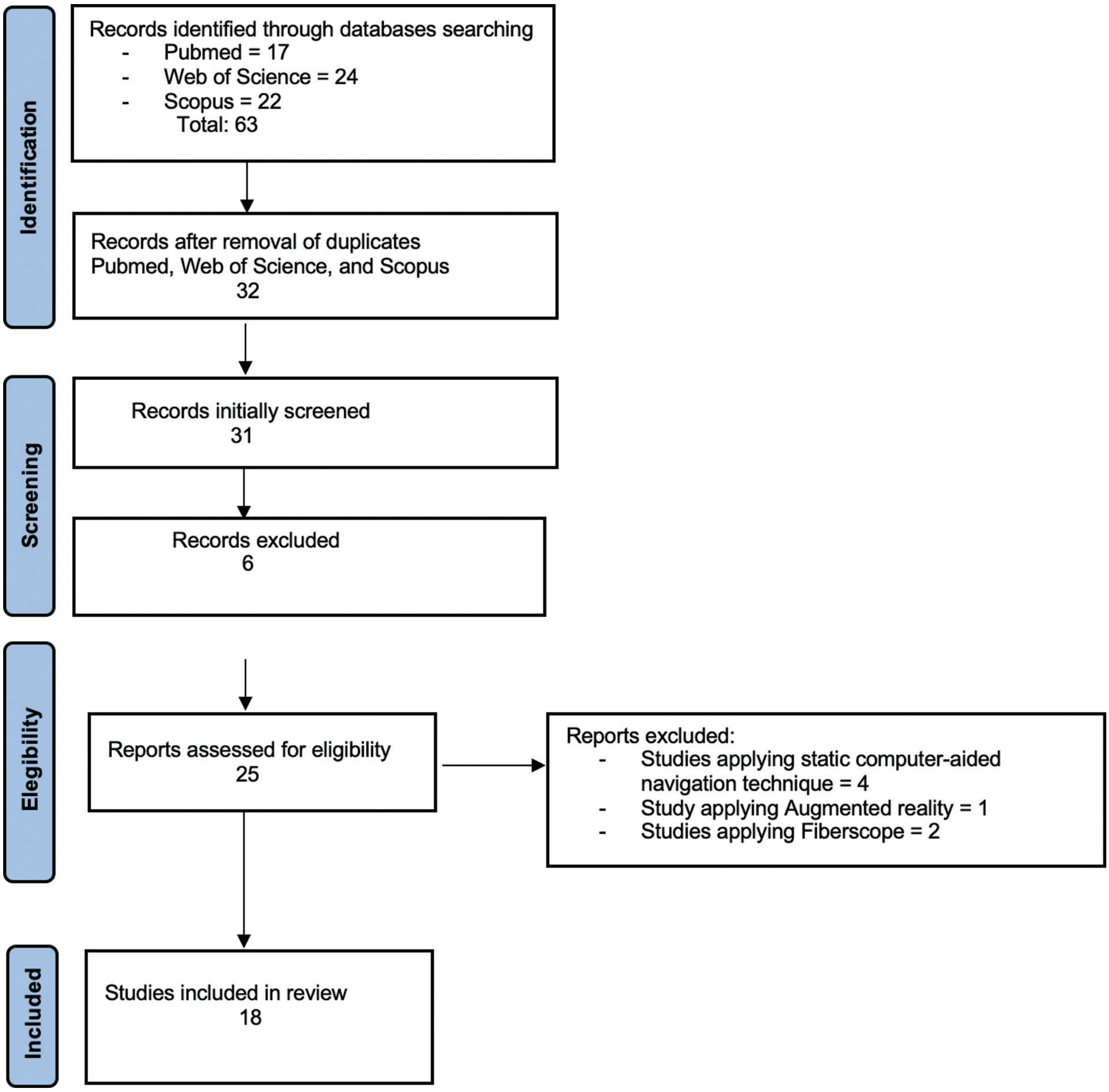

Supplementary Fig. S1 PRISMA flow diagram.

 © 2022. The Author(s).

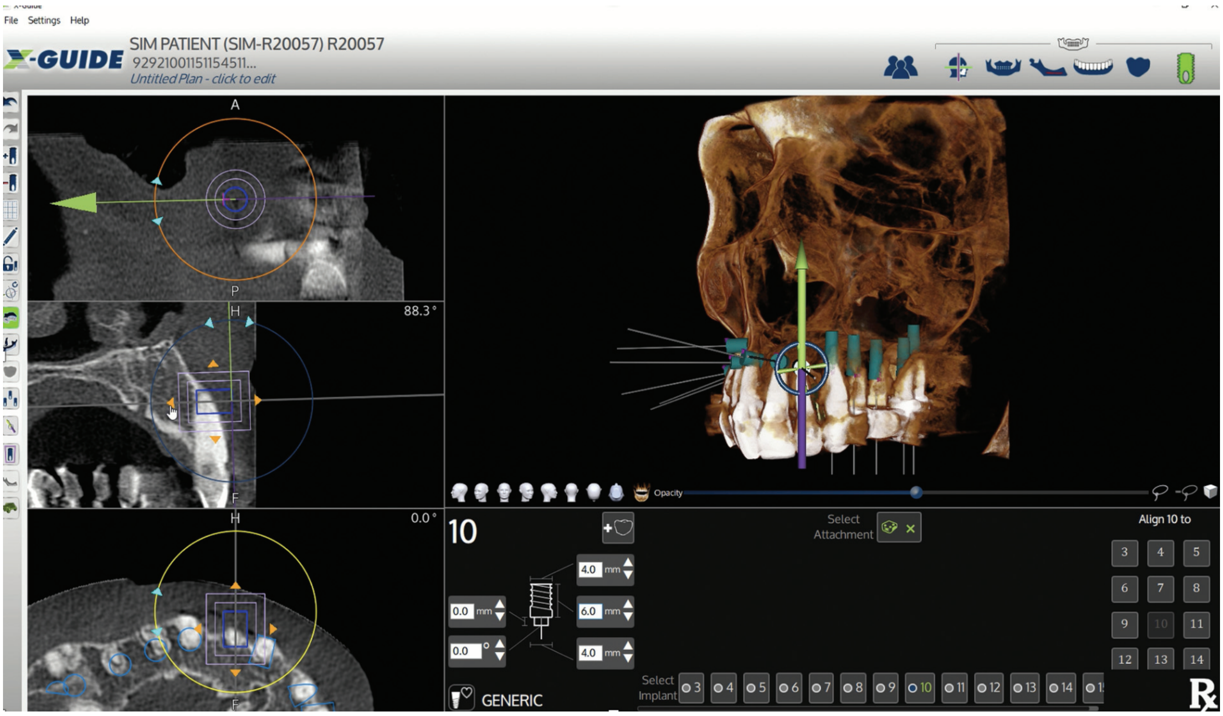

Supplementary Fig. S2 Planning endodontic microsurgery on dynamic navigation system (DNS) software (X-Guide's Implant Planning Software).

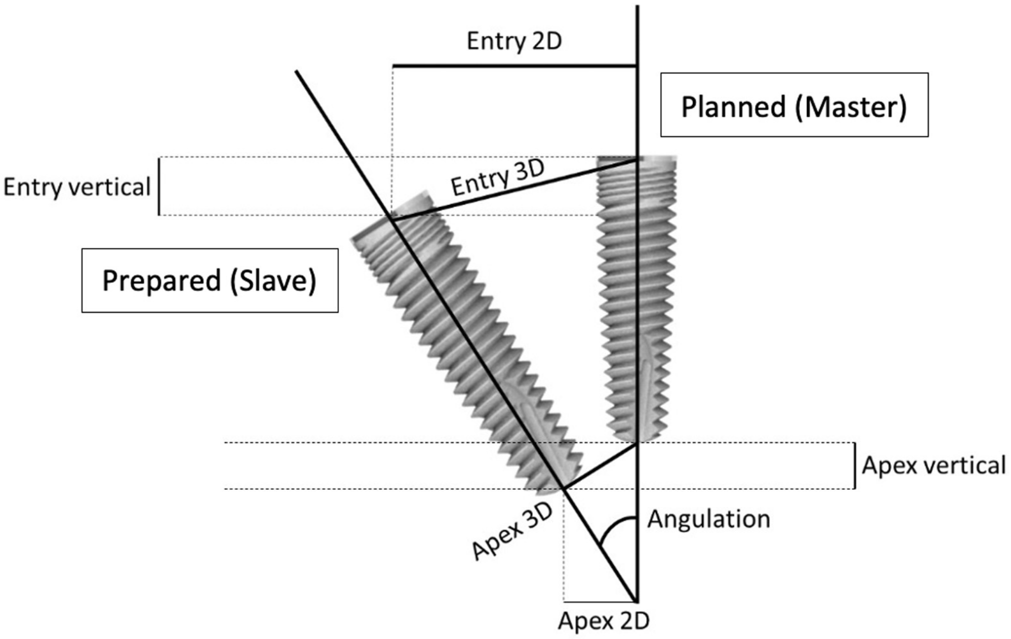

Supplementary Fig. S3 Deviation metrics: the deviation between the planned position and the final position. 2D: two dimensions (lateral); 3D: three dimensions (global). 1. *Entry (2D) lateral*: the deviation between the planned position and the final position in the "x" and "y" dimensions of space in an occlusal view, in millimeters (mm); 2. *Entry (3D) global*: the deviation between the planned position and the final position in three dimensions of space "x," "y," and "z"-axis, in mm; 3. *Entry depth*: vertical distance (depth) between the planned and final positions "z," in mm; 4. *Apex (2D) (lateral)*: the deviation between the planned position and the final position of the apex in the "x" and "y"-axis dimensions of space in an occlusal view, without taking deviation in-depth "z"-axis into account, in mm; 5. *Apex (3D) global*: the deviation between the planned position and the final position of the apex in three dimensions of space "x," "y," and "z"-axis, in mm; 6. *Apex depth*: vertical distance (depth) between the planned position and the final position of the apex; and 7. *Angulation*: the angular deviation between the central axes of the planned position and the final position, in sexagesimal degree (°).

 © 2022. The Author(s).

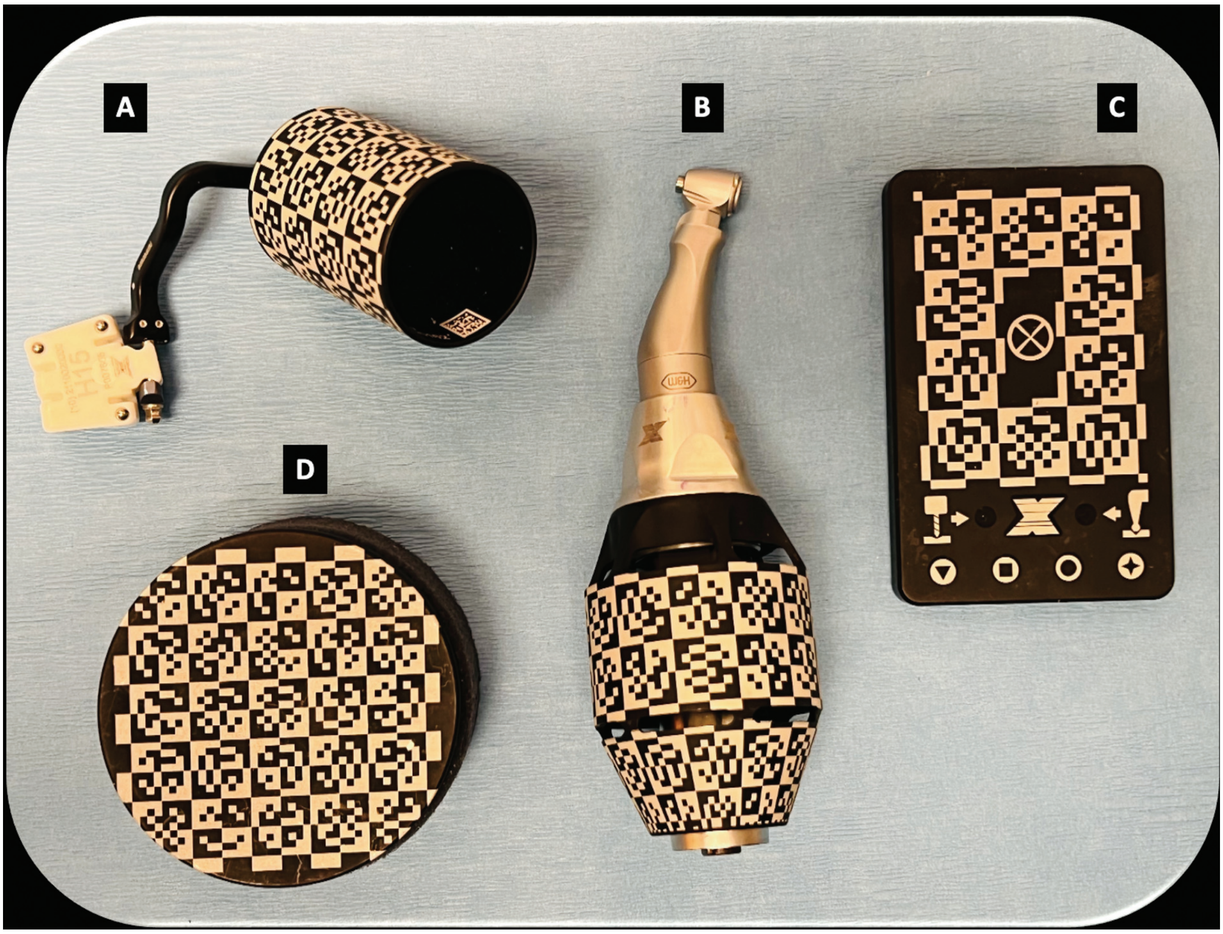

Supplementary Fig. S4 Dynamic navigation system (DNS) tools: **(A)** Patient's head/mouth tracker tool + X-clip; **(B)** Handpiece tracker tool + Handpiece; **(C)** Drill calibration tool; **(D)** Handpiece calibration tool.

 © 2022. The Author(s).
